# Semaglutide Treatment of Excessive Body Weight in Obese PCOS Patients Unresponsive to Lifestyle Programs

**DOI:** 10.3390/jcm12185921

**Published:** 2023-09-12

**Authors:** Enrico Carmina, Rosa Alba Longo

**Affiliations:** Endocrinology Unit, University of Palermo School of Medicine, 90144 Palermo, Italy

**Keywords:** PCOS, obese PCOS, semaglutide, weight loss, impaired fasting glucose, insulin resistance

## Abstract

In spite of the widespread use of lifestyle modifications programs, many patients with PCOS are obese and prevalence of obesity in PCOS remains high. In this study, we present the data on the use of semaglutide, an incretin mimetic drug, in obese PCOS patients who were unresponsive to a lifestyle modification program. Twenty-seven obese patients with a diagnosis of PCOS, who did not reduce their body weight by a lifestyle modification program, were included in this study and treated by semaglutide, 0.5 mg subcutaneously once a week. After three months of treatment, an improvement in body weight with a mean decrease in body weight of 7.6 kg and a mean BMI loss of 3.1 was observed, while very few side effects were reported. Almost 80% of the studied obese PCOS patients obtained at least a 5% decrease in their body weight. Only a few patients (22%) obtained a decrease in body weight lower than 5% and were considered non-responsive to semaglutide, at least at the used doses. These patients presented a more severe obesity than responsive patients. Independently of results on body weight, and in patients who did not obtain a 5% decrease in their body weight, insulin basal values decreased, and HOMA-IR improved. Fasting blood glucose normalized in 80% of semaglutide-treated IFG PCOS women. In patients who were responsive to semaglutide (weight loss > 5%), the treatment was continued for additional three months. Weight loss slowed but continued and, at the end of the six months of therapy, the mean body weight loss was 11.5 kg and mean BMI reduced from 34.4 to 29.4. A total of 80% of responsive patients normalized menstrual cycles. In conclusion, treatment with semaglutide, at low doses, significantly reduces body weight in almost 80% of obese PCOS patients who were unresponsive to a previous lifestyle plan. It is often associated with the normalization of menstrual cycles, and these important results are obtained with very few side effects.

## 1. Introduction

Obesity is common in polycystic ovary syndrome (PCOS) [1,2,3] and is associated with severe insulin resistance, metabolic alterations and cardiovascular complications [2,3,4,5,6]. It strongly affects the long-term prognosis of PCOS patients [2,3,6]. In obese PCOS patients, lifestyle modification programs that include diet and physical exercise are considered first-line treatment and many studies have shown that these programs may improve the clinical presentation and long-term prognosis of the syndrome [2,3]. However, many patients with PCOS remain obese, and the prevalence of obesity in PCOS is higher than in the general population [1,2,3,6].

In the past, drugs that improve insulin resistance, like metformin, have shown only a modest ability to improve body weight in obese PCOS patients [2,7,8]. Better results have been obtained with products that mimic intestinal incretins and, in particular, the glucagon-like peptide 1 (GLP-1) effect, improving not only insulin production but also reducing appetite and energy intake [9]. Liraglutide, a GLP-1 mimetic product, may reduce body weight in obese subjects [10] and has been used in obese PCOS patients with mixed results [11,12]. Most meta-analyses report that many obese PCOS patients treated by liraglutide lose more than 5% of their body weight, with a total weight loss of 4–6 kg [11]. These results are not really satisfactory because most patients remain obese, and side effects are common and determine a high withdrawal rate [11,12].

More recently, semaglutide, a new incretin mimetic product, has shown the ability to more consistently reduce body weight in obese type 2 diabetic patients as well as in obese nondiabetic subjects [12,13,14,15,16,17]. However, data on obese PCOS patients are almost non-existent and it is unclear whether semaglutide may be used to reduce body weight in this disorder and what doses of the drug should be used to avoid side-effects.

In this study, we present data on use of low doses (0.5 mg once a week subcutaneously) of semaglutide in 27 obese PCOS patients who were unresponsive to a lifestyle modification program. Our data show that this treatment reduces body weight in most obese PCOS patients.

## 2. Materials and Methods

Twenty-seven obese (body mass index (BMI) kg/m^2^, ≥30) patients with a diagnosis of PCOS who were unresponsive to a lifestyle modification program were included in this study. All these patients were referred between 2021 and 2022 because of obesity, hyperandrogenism and menstrual disorders, and were initially treated for three months with a lifestyle program (low-fat hypocaloric diet, a personalized program of physical exercise and psychological support) but did not reduce body weight (body weight loss lower than 5%). 

The diagnosis of PCOS was based on Rotterdam criteria, with two out of three of the following criteria: chronic anovulation, clinical or biologic hyperandrogenism, and/or polycystic ovaries on ultrasound after the exclusion of other medical disorders [2]. All studied patients presented phenotype A PCOS (chronic anovulation, hyperandrogenism and polycystic ovaries) [2,4].

In all patients, serum levels of total testosterone (T), 17-hydroxy-progesterone (17OHP), fasting insulin and fasting glucose were determined on days 3–5 of the cycle. An oral glucose test (OGTT, 75 g of glucose) was performed with measurement of blood glucose at 30, 60 and 120 min. In non-menstruating women, blood samples were obtained after withdrawal bleeding after progestogen administration. 

Anovulation was defined as serum progesterone < 3 ng/mL (<9.54 nmol/L). Clinical hyperandrogenism was defined as the presence of hirsutism. Hirsutism was assessed by Ferriman–Gallwey–Lorenzo scores [18], and patients with scores higher than 6 were considered hirsute. Adult acne and female-pattern hair loss were not considered a sign of hyperandrogenism if androgen levels were normal [19,20]. Biochemical hyperandrogenism was defined as serum testosterone > 34 ng/dL. 

Total testosterone was determined by mass spectrometry after liquid chromatography (LC/MS) assay while 17OHprogesterone and serum insulin were measured by specific RIAs using previously described methods [21,22]. Insulin sensitivity was evaluated by the quantitative HOMA-IR method (glucose mg/dL × insulin mU/mL/405) [23]. In all patients, serum 17OH progesterone values were determined to exclude the existence of Non-Classic Congenital Adrenal Hyperplasia [24]. In some patients, because of clinical suspicion, urinary free cortisol, and serum prolactin and TSH were measured by commercial RIA methods to exclude other endocrine conditions.

In all assays, intra-assay and inter-assay coefficients of variation did not exceed 6% and 15%, respectively. 

Transvaginal pelvic ultrasound was performed using a transducer frequency of 8–10 MHz and the presence of polycystic ovaries was established by the finding of an increased number of follicles, each of which measured 2–10 mm in diameter, and/or increased ovarian size [25]. 

No patient had received any medication for at least 3 months before the study, and all patients gave informed consent for this evaluation. The treatment with semaglutide and the research protocol obtained institutional approval from the ethical committee of our university (2021/22). 

The various values of the women with PCOS were compared to those of 65 normal ovulatory women. These controls were drawn from the same population and did not report complaints of hyperandrogenism or menstrual irregularities.

All studied obese PCOS patients were treated by semaglutide, 0.5 mg subcutaneously once a week for three months. During this period, side effects were recorded each month while, at the end of the three months of treatment, body weight, BMI and FGL scores were reassessed, fasting blood glucose and serum insulin were re-evaluated and HOMA-IR was calculated again. 

In responsive patients (weight loss > 5%), the semaglutide treatment was prolonged at the same dose for an additional three months and, at the end of the six months of therapy, body weight, BMI, FGL scores, fasting blood glucose and serum insulin were re-assessed and menstrual cycle characters and side effects were recorded.

### Statistical Analysis

Statistical analyses were performed using Statview 5.0 (SAS Institute, Cary, NC, USA). Because several values were not normally distributed, a log transformation was necessary to obtain a normal distribution. Analysis of variance (ANOVA), followed by Tukey tests, were performed to assess differences in clinical and biochemical parameters between basal conditions and three and six months of treatment with semaglutide. The accuracy of parameters used to discriminate between basal and post-treatment values were evaluated using ROC curve analyses. Differences in reliability between different parameter values were assessed by Tukey multiple comparison tests. *p* < 0.05 was considered statistically significant. All results are reported as mean ± SD. 

## 3. Results

All studied PCOS patients were obese (BMI range 30.4–47.9) and 33% of patients presented moderate (BMI > 35 < 40) or severe (BMI > 40) obesity. As shown in Table 1, the studied cohort of PCOS patients had significantly (*p* < 0.01) increased values of BMI, FGL scores, total testosterone, 17OH Progesterone, fasting glucose, insulin, HOMA-IR and triglycerides and significantly (*p* < 0.01) lower levels of HDL-cholesterol compared to normal controls. No differences in total cholesterol and LDL cholesterol values between patients and controls were observed. All studied patients reported irregular (oligomenorrhea or secondary amenorrhea) menses.

Fifteen PCOS patients (55.6%) had fasting values of blood glucose higher than 100 mg/dL (IFG, increased fasting glucose, patients), while four patients (all with increased fasting glucose) presented impaired glucose tolerance (blood glucose > 140 mg/dL and <200 mg/dL at 120 min after OGTT). No patients were affected by type 2 diabetes.

After semaglutide treatment for three months, BMI, body weight, fasting glucose, insulin and HOMA-IR values significantly (*p* < 0.01) decreased. The mean weight loss was 7.6 ± 3 kg (range 2–12 kg) and mean BMI loss was 3.1 ± 1 (range 0.9–4.6). % body weight loss was 8.9 ± 3.7 (range 2.8–14.6%). Fasting glucose, insulin and HOMA-IR decreased significantly (*p* < 0.01) and twelve IFG PCOS patients (80%) showed normal fasting blood glucose, while in the remaining three IFG patients (20%) the values of fasting glucose improved (decrease of at least 10 mg/dL) but remained higher than 100 mg/dL. 

% weight loss negatively correlated with basal BMI (*p* < 0.01) and basal body weight (*p* < 0.05) but not with basal blood glucose or insulin or HOMA-IR values. No correlations were found between decrease in fasting glucose or insulin or calculated HOMA-IR and basal BMI, body weight, glucose, insulin, or HOMA-IR. 

Six patients (22.2%) lost less than 5% of their body weight and were considered unresponsive to semaglutide, while twenty-one patients lost more than 5% of their body weight and were considered responsive to semaglutide. Nine responsive patients lost more than 10% of their body weight and were considered highly responsive to semaglutide. In Table 2, some characters of highly responsive and non-responsive PCOS patients were compared. PCOS patients who did not respond to semaglutide presented with a significantly (*p* < 0.01) higher BMI than PCOS patients who lost > 10% of their body weight after treatment with semaglutide.

Twenty-one obese PCOS patients who were responsive (weight loss > 5%) to semaglutide therapy continued the treatment with this product at the same dose for an additional three months. In Table 3, the results of the treatment on BMI, and glucose metabolism are shown. In these responsive PCOS patients, the additional three months of treatment with semaglutide induced a small further decrease in body weight (mean weight loss −2.5 kg) with a total weight loss of 11.5 kg after six months of therapy (Table 3). No further changes in fasting glucose, insulin or HOMA-IR were found. In these responsive patients, menstrual disorders improved, with fifteen PCOS women (71% of responsive patients) achieving normal menses. No significant changes in FGL scores were observed.

Treatment with semaglutide, 0.5 mg once a week for up to six months induced few side effects, with nine patients (33%) reporting morning nausea and two patients complaining of sporadic vomiting. No patient withdrew from the therapy because of side effects.

## 4. Discussion

In this study, we evaluated the effect of low doses (0.5 mg subcutaneously once a week) of semaglutide on body weight and insulin and glucose blood levels in 27 obese PCOS women who were unresponsive to a lifestyle program. All PCOS patients presented a classic form of PCOS (phenotype A: chronic anovulation, hyperandrogenism and polycystic ovaries) and were treated by 0.5 mg of semaglutide subcutaneously once a week. No specific lifestyle plan was added to the pharmacologic treatment, but patients were told to maintain a normal food intake and a regular physical activity. 

Our results show an improvement in body weight, with a mean decrease in body weight of 7.6 kg and a mean BMI loss of 3.1. Almost 80% of obese PCOS patients who were unresponsive to a lifestyle plan, obtained an at least 5% decrease in their body weight and this was associated with a significant improvement in basal glucose and insulin resistance (calculated by HOMA-IR). Only a few patients (22%) showed a decrease in body weight of lower than 5% and were considered non-responsive to semaglutide, at least at the used doses.

The mean weight loss observed after treatment with semaglutide was larger than that reported with metformin [2,7,8] or liraglutide [9,10,11,12] and was obtained using low doses of the product with very few side effects. Independently of results on body weight, and also in patients who did not reach a 5% decrease in their body weight, insulin basal values decreased, and HOMA-IR improved in all treated patients. Fasting blood glucose normalized in 80% of semaglutide-treated IFG PCOS women, with the remaining few IFG PCOS patients obtaining a decrease of at least 10 mg/100 mL of their fasting blood glucose. This suggests that semaglutide, independently of its effect on body weight, may represent a good alternative to metformin for improving insulin resistance and preventing type 2 diabetes in PCOS.

Interestingly, comparing patients who were unresponsive to semaglutide therapy with patients who were highly responsive (weight loss > 10%), we found that unresponsive patients were significantly more obese (mean BMI 40 versus 32, *p* < 0.01). This may suggest that, in severely obese patients, higher doses of semaglutide are needed. Consistently with this, in most studies on the general population, higher doses of semaglutide (1 mg or more once a week) have been used for the treatment of obesity [13,14,15,16,17]. However, it should be noted that many patients with severe obesity present a genetic form of obesity that may not be sensitive to drugs that mimic an incretin effect [26,27].

In patients who were responsive to semaglutide (weight loss > 5%), the treatment was continued for an additional three months. Weight loss slowed but continued and, at the end of the six months of therapy, the mean body weight loss was 11.5 kg and mean BMI reduced from 34.4 to 29.4. This very good treatment result was associated with an improvement in menstrual cycles, which, in almost 80% of the responsive patients, became normal. All this was obtained with very few side effects.

Of course, other studies are needed, but these initial results look very promising and suggest that semaglutide may become a very important tool for the treatment of obese PCOS patients.

In conclusion, treatment with semaglutide at low doses significantly reduces body weight in almost 80% of obese PCOS patents who were unresponsive to a previous lifestyle plan. It is often associated with the normalization of menstrual cycles and these important results are obtained with very few side effects. The best results are obtained in patients with mild obesity, while patients with severe obesity are generally unresponsive to the product, at least at the used doses. Independently of the effects on body weight, semaglutide treatment improves insulin resistance and may normalize fasting glucose in IFG PCOS patients. 

## Figures and Tables

**Table 1:** Some clinical and hormonal data of 27 obese PCOS patients and 65 normal controls.

	PCOS Patients	Controls
Age (yrs.)	30 ± 9	29 ± 3
BMI (kg/m^2^)	35.7 ± 6 **	23 ± 4
FGL index	10 ± 2 **	3 ± 1
LH/FSH ratio	1.7 ± 0.5 **	1.1 ± 0.4
Total testosterone (ng/dL)	53 ± 15 **	22 ± 5
17-OH-Progesterone (ng/mL)	1.2 ± 0.4 **	0.8 ± 0.2
Fasting glucose (mg/dL)	98 ± 12 **	78 ± 9
Insulin (mU/mL)	16 ± 7 **	9 ± 3
HOMA-IR	4 ± 2 **	1.2 ± 0.3
Total Cholesterol (mg/dL)	171 ± 22	168 ± 20
HDL Cholesterol (mg/dL)	46 ± 8 **	55 ± 5
LDL Cholesterol (mg/dL)	108 ± 17	105 ± 15
Triglycerides (mg/dL)	94 ± 21 **	65 ± 18

** *p* < 0.01 versus controls.

**Table 2:** Basal BMI, fasting glucose, insulin and HOMA-IR in PCOS patients treated by semaglutide and divided according to their response to the therapy.

	Highly Responsive PCOS Patients (Weight Loss > 10%)	Non-Responsive PCOS Patients (Weight Loss < 5%)
Number of patients	9	6
BMI (kg/m^2^)	32 ± 5 **	40 ± 5
Fasting glucose (mg/dL)	94 ± 11	102 ± 11
Insulin (mU/mL)	14.9 ± 2.9	17.8 ± 10
Homa-IR	3.6 ± 0.5	4.6 ± 2

** *p* < 0.01 versus non responsive PCOS patients.

**Table 3:** Changes in BMI, body weight, fasting glucose, insulin, and insulin resistance (HOMA-IR) (mean ± SD) in 21 obese PCOS women responsive (weight loss > 5%) to semaglutide treatment (0.5 mg subcutaneously once a week).

	Basal	After 3 Months of Treatment with Semaglutide	After 6 Months of Treatment with Semaglutide
BMI (kg/m^2^)	34.4 ± 5.9	30.8 ± 5 **	29.4 ± 5 **
Body weight (kg)	85 ±15	76 ± 16 **	73.5 ± 15 **
Fasting glucose (mg/dL)	97 ± 12	90 ± 8 **	90 ± 6 **
Insulin (mU/mL)	17 ± 7	11 ± 5 **	11 ± 5 **
HOMA-IR	3.5 ± 2	2.5 ± 1 **	2.4 ± 0.8 **

** *p* < 0.01 versus basal values.

## Data Availability

Data supporting results can be found at the office of Prof. Carmina.
